# CoQ10-related sustained remission of proteinuria in a child with COQ6 glomerulopathy—a case report

**DOI:** 10.1007/s00467-018-4083-3

**Published:** 2018-09-19

**Authors:** Małgorzata Stańczyk, Irena Bałasz-Chmielewska, Beata Lipska-Ziętkiewicz, Marcin Tkaczyk

**Affiliations:** 1 Department of Pediatrics, Immunology and Nephrology, Polish Mother’s Memorial Hospital Research Institute of Lodz, Rzgowska St. 281/289, 93-338 Lodz, Poland; 2 Department of Nephrology and Hypertension of Children and Adolescents, Medical University of Gdansk, Gdansk, Poland; 3 Department of Biology and Medical Genetics, Clinical Genetics Unit, Medical University of Gdansk, Gdansk, Poland; 4 Department of Pediatrics, Immunology and Nephrology, Polish Mother’s Memorial Hospital Research Institute of Lodz, Division of Didactics in Pediatrics, Medical University of Lodz, Lodz, Poland

**Keywords:** Nephropathy, Primary coenzyme Q10 deficiency, Ubiquinone, Treatment, Genetics, Child

## Abstract

**Background:**

Treatment of steroid resistant nephrotic syndrome is still a challenge for physicians. There are a growing number of studies exploring genetic background of steroid-resistant glomerulopathies.

**Case diagnosis/treatment:**

We present the case of a 4-year-old girl with steroid-resistant glomerulopathy due to a COQ6 defect with no additional systemic symptoms. The disease did not respond for second-line therapy with calcineurin inhibitor, but it remitted completely after oral treatment with 30 mg/kg/d of coenzyme Q10 (CoQ10). The patient was identified to be a compound heterozygote for two pathogenic variants in COQ6 gene: a known missense substitution c.1078C > T (p.R360W) and a novel frameshift c.804delC mutation. After 12 months of CoQ10 therapy, the child remains in full remission, her physical development accelerated, frequent respiratory airways diseases subsided.

**Conclusions:**

Genetic assessment of children with steroid-resistant nephrotic proteinuria enables therapy optimization. Proteinuria caused by a COQ6 gene defect can be successfully treated with CoQ10.

## Introduction

Treatment of steroid-resistant nephrotic syndrome is still a challenge for physicians around the world, despite the progress in immunosuppressive treatment methods. Even without severe symptoms of nephrotic syndrome, significant proteinuria can be a risk factor of accelerated kidney damage [1]. Second-line therapy is fraught with high risk of side effects and is sometimes inefficacious. There are a growing number of studies exploring the genetic background of steroid-resistant glomerulopathies [2, 3]. Reports of coenzyme Q complex defects have recently been published. These include patients with mutations in *COQ2*, *COQ6*, *PDSS2* and *COQ8B*. A few families with *COQ6* mutations with multiple organ involvement were described in which coenzyme Q10 (CoQ10) therapy was successfully carried out [4, 5] (Table 1). We present the case of steroid resistant glomerulopathy due to a *COQ6* defect with no additional systemic symptoms, which responded to oral CoQ10 treatment.

**Table 1:** Patients with *COQ6* mutations treated with CoQ10 reported to date

Nucleotide mutation	Exon (segregation)	Protein change	Age at onset	Kidney disease	Origin	Histology	CoQ10 start	Treatment/CoQ10 dose	Response	Extrarenal findings	Reference
c.763G > A	7 (hom)	p. Gly255Arg	3 mo	SRNS	Turkey	NDA	NDA	CoQ10: 100 mg/d	–SND improvement –ESRD (4 mo)	SND facial dysmorphism	[4]
c.763G > A	7 (hom)	p. Gly255Arg	2 mo	proteinuria	Turkey	NDA	2 mo	CoQ10: 15 mg/kg/d → 30 mg/kg/d after 2 months of treatment ACE-I: 1.25 mg/d	–uPCR: 40 mg/mg → 8 mg/mg (after 2 mo) → 5.8 mg/mg → 4.8 mg/mg (15 mo) → 0.55 mg/mg (4.5 y.) –Normal renal function	Asymptomatic at the onset SND (10 mo) Bilateral nephrolithiasis (5 mo) GR (10 mo)	[4]
c.782C > T	7 (hom)	p.Pro261Leu	8 mo	SRNS	Italy	MPGN	NDA	CoQ10 – to prevent neurological symptoms; dose – NDA	–ESRD at 20 mo –Lack of neurological symptoms	None	[5]
c.1058C > A	9 (hom)	p.Ala353Asp	2.5 yr	SRNS	Turkey	FSGS	5.5 y	CoQ10 –dose: NDA CsA – dose:NDA	24 h protein in urine: 7 mg/m2/h → 3.7 mg/m2/h (after 2 mo) → full remission –No hearing improvement –Relapse of proteinuria 57 mg/m2/h after cessation of CoQ10	SND	[4, 6]
c.1078C > T	9 (hom)	p. Arg360Trp	10 mo	nephrotic proteinuria	China	NDA	NDA	CoQ10 – dose: 30 mg/kg	–uPCR 7.2 mg/mg → 1.3 mg/mg (after 2 mo) → 0.01 mg/mg (after 3 mo) –Improvement of growth retardation–SND (2 yr)	Cardiovascular abnormality Motor and mental retardation Unilateral ptosis	[7]

*SRNS* steroid resistant nephrotic syndrome, *FSGS* focal segmental glomerulosclerosis**,** *MPGN* membranoproliferative glomerulonephrirts, *CoQ10* coenzyme Q10, *ACE-I* angiotensin-converting-enzyme inhibitors, *CsA* cyclosporine A*, SND* sensorineural deafness, *ESRD* end-stage renal disease, *uPCR* urine protein-creatine ratio, *GR* growth retardation, *NDA* no data available, *hom* homozygous in affected individual

## Case study

A 4-year-old girl (A.C.) of non-consanguineous parents was diagnosed with glomerulopathy at 2 years of age. The onset of the disease was associated with febrile pharyngitis. Diagnostics revealed nephrotic proteinuria (57 mg/kg/d) with hypercholesterolemia, but without hypoalbuminemia or peripheral oedema.

**Fig. 1:**
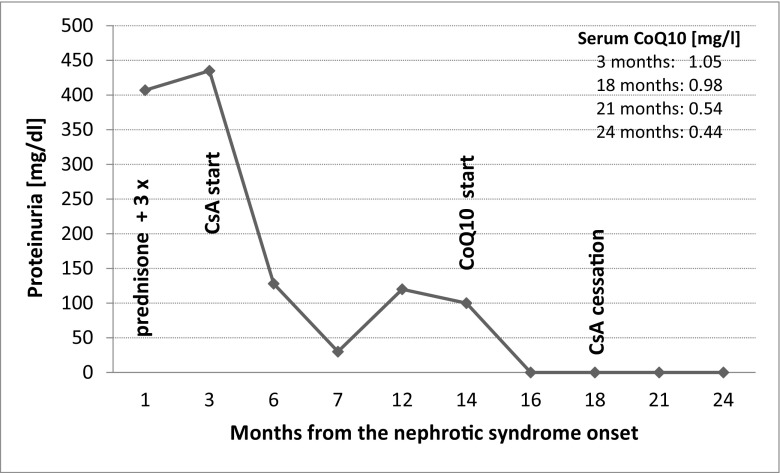
Changes of proteinuria and CoQ10 serum concentration (right upper part of the chart) in the course of treatment

The first-line therapy with an angiotensin converting enzyme (ACE) inhibitor (ramipril 1.25 mg/d) and daily prednisone (60 mg/m^2^) was started, giving no response for 4 weeks. Afterwards, 3 pulses of methylprednisone were administered, also without effect. Kidney biopsy showed focal segmental glomerular sclerosis and minimal change (FSGS/MCNS). Due to the lack of treatment effect the girl was qualified to second-line therapy with a calcineurin inhibitor—cyclosporine A (CsA). Only partial remission was achieved (Fig. 1). Proteinuria rose constantly during frequent (once a month) respiratory tract infections. The child was growing poorly, not achieving relevant body weight and height gains. She also had symptoms of mild but difficult to treat caries. Biochemical side effects of CsA developed—we observed slight elevation of serum uric acid (6.3 mg/dl) and urea (39 mg/dl), and hypercholesterolemia did not fully subside. Blood pressure was mildly elevated.

The patient underwent two-step genetic testing. Mutational screening of the entire coding region of *NPHS2* and exons 8,9 of *WT1* performed by Sanger sequencing was followed by custom-targeted amplicon-based multi-gene NGS panel analysis (Multiplicom, Niel, Belgium) for FSGS and related glomerulopathies. NGS findings were verified by Sanger sequencing, which was also used to confirm the presence of mutations in *trans* through parental segregation. The patient was identified to be a compound heterozygote for two pathogenic variants in the *COQ6* gene: a known missense substitution c.1078C > T (p.R360W) and a novel frameshift c.804delC mutation (Table 2).

**Table 2:** *In-silico* characteristics of the detected *COQ6* pathogenic variants

Variant (rs ID if applicable)	Exon	Protein change	Conservation	Prevalence in control population [MAF]*	Grantham difference score	Human Splicing Finder 3.0	PolyPhen2 SIFT, CADD Predictors
c.804delC	8	p.Leu269Trpfs*13	Residue conserved, within conserved region	Not reported	n/a	Activation of an exonic cryptic donor site. Creation of an exonic ESS site	n/a
c.1078C > T rs778856227	9	p. Arg360Trp	Residue conserved, within conserved region	Singleton	101	Creation of an exonic ESS site	Deleterious (PolyPhen 1.0; SIFT 0.02, CADD 35)

Reference seq. ENST00000334571; NM_182476.2; UniProt peptide Q9Y2Z9.

Estimation based on data of 60,706 multiethnic individual genomes catalogued by the Exome Aggregation Consortium (ExAC; accessed 28th May 2018)

*MAF* minor allele frequency, *n/a* not applicable, *ESS* exonic splicing silencer

Other organ involvement described in patients with *COQ6* mutations was evaluated. The hearing deficit, encephalopathy, seizures, ataxia, and facial dysmorphism were excluded by specialist assessment.

After genetic diagnosis was set, we introduced into the therapy 30 mg/kg/d CoQ10 (ubiquinone). Within 1 month, the girl was in complete remission, which enabled the reduction and discontinuation of CsA after 3 months.

After 12 months of CoQ10 therapy, even though the serum CoQ10 level is variable, the child is still in remission. She does not suffer from any infections despite attending kindergarten. We observed growth acceleration (3.4 kg and 13 cm in 12 months) and improvement of teeth condition, blood pressure dropped to normal values, and biochemical parameters were again within reference ranges (partially due to CsA cessation). The dose of CoQ10 was adjusted to the degree of proteinuria rather than weight.

## Discussion

Coenzyme Q10 is involved in the mitochondrial oxidative phosphorylation chain, providing energy for cells and antioxidant protection to plasma membranes and lipoproteins [8]. It also takes part in the prevention of cell apoptosis [9]. Primary deficiency caused by mutations in *COQ* genes results in various manifestations and heterogenic symptoms from kidneys and the nervous and muscular systems [10]. Among the so far studied genes, 4: *PDSS2*/*COQ1*, *COQ2*, *COQ6*, and *ADCK4*/*COQ8B* are associated with steroid-resistant nephrotic syndrome (SRNS)—they are mutated in about 1% of cases [2, 3]. Mutations in *PDSS2* and *COQ2* are also mostly associated with progressive encephalopathy, seizures, and hypertrophic cardiomyopathy, while *ADCK4* disease typically manifests as an isolated nephropathy [11].

Most of the so far described individuals with bi-allelic mutations in *COQ6* present sensorineural deafness (SND) and progressive SRNS, and almost half of them die in early childhood [4, 6, 12]. Other described concomitant symptoms are ataxia, seizures, facial dysmorphism, kidney stones, and growth retardation. Among children treated with CoQ10, in one (homozygote c.763G > A (p.G255R)) proteinuria decreased but without complete remission, and it did not prevent the development of sensorineural deafness (SND) or growth retardation. In another (another homozygote c.763G > A (p.G255R)), treatment resulted in improvement of SND. In a third one (homozygote c.1058C > A (p.A353D), proteinuria was completely reduced, but the treatment did not improve hearing loss.

Chinese authors reported a patient with the same variant *COQ6* c.1078C > T (p.R360W) as in our patient but present in a homozygous state who presented nephrotic proteinuria and motor and mental retardation with ptosis and cardiovascular abnormalities. He was successfully treated with 30 mg/kg of CoQ10. Despite total remission of proteinuria, at the age of 2 years he developed SND [7].

Not only did our patient lacked extrarenal symptoms commonly associated with COQ6 mitochondropathy, but also responded very well to the therapy. Not only did the proteinuria remit, but the girl’s growth velocity improved and caries subsided. It might be hypothesised that body weight gain is an effect of the absence of frequent infections and lack of urine protein loss. Improvement of oral cavity health could probably be an effect of the anti-inflammatory and anti-oxidative action of coenzyme Q. Some data show that conditions associated with chronic inflammation of low grade respond well to CoQ, with a significant decrease in TNFα plasma levels without having an effect on CRP and Il-6 production [13]. There is some evidence that oral diseases, i.e., dental carries, are also associated with oxidative stress [14].

The interesting finding in the present case is that although the girl was treated with daily 30 mg/kg of CoQ10, there was no increase in CoQ10 plasma concentration, and it was even lower after 6 months despite clinical remission of glomerulopathy. The lack of increase of plasma level of CoQ10 during therapy in our patient is unclear. Perhaps the local action of CoQ10 is more important in this case. Due to the lack of correlation between the dose of CoQ10, its serum level and clinical improvement we postulate not to assess CoQ10 concentration as an indicator of successful treatment.

Our patient apart from nephrotic range proteinuria did not present other described vastly in the literature extrarenal symptoms. In clinical practice physicians have to manage many children with proteinuria as the only symptom, and the treatment in many cases is ineffective and even loaded with side effects. It raises the question: if we have overlooked some patients with genetic defects who could be treated more successfully after setting the proper diagnosis of the cause of proteinuria. It can be hypothesised that in patients with refractory proteinuria of unknown origin early genetic screening for treatable defects such as presented could provide a highly practical tool for the therapy optimization, before the progression of kidney damage. The PodoNet consortium revealed a number of such Janus-faced genes presenting in an oligosyndromic manner (including *WT1*, *SMARCAL1*, *ADCK4/COQ8B*, *COL4A3-5*, *CLCN5*), where incidental diagnosis through NGS screening allowed for identification of the causative mutation in patients lacking extrarenal manifestation(s) classically associated with the particular genetic defect [3].

Going further, one could even consider an empiric therapy with CoQ10 in all steroid-resistant nephrotic syndrome patients. On the basis of the present case, already a short, lasting 2–3 months initial course of therapy would give the evidence about its effectiveness. Such a quick response for treatment was reached by others in patients with *COQ8B* defect [11]. Considering that CoQ10 therapy generally comes up with mild side effects, the treatment results could quickly provide a justification for such an approach [15]. CoQ10 oral formulations are diet supplements. Known side effects include stomach discomfort, loss of appetite, nausea, vomiting, diarrhoea, allergic skin reactions and lowering of blood pressure. Although it is promising, more clinical data about its effectiveness and safety are needed to determine an algorithm when to suspect *COQ6* defect and in which cases to treat proteinuric patients with CoQ10.

## Summary

Genetic assessment of children with steroid-resistant nephrotic proteinuria enables therapy optimization. Prompt and clear diagnosis of the cause of proteinuria is of the highest importance enabling successful treatment of proteinuria, and hopefully avoiding progression to end-stage renal disease. Proteinuria caused by *COQ6* gene mutation can be successfully treated with CoQ10.
